# Comprehensive analysis of lymph nodes metastasis associated genes in cervical cancer and its significance in treatment and prognosis

**DOI:** 10.1186/s12885-021-08945-8

**Published:** 2021-11-16

**Authors:** Ping Yang, Youqin Ruan, Zhiling Yan, Yang Gao, Hongying Yang, Shaojia Wang

**Affiliations:** Department of Gynecology, The Third Affiliated Hospital of Kunming Medical University, Yunnan Cancer Hospital, Yunnan Cancer Center, Kunming, 650118 China

## Abstract

**Background:**

Cervical carcinoma is one of the most common malignant tumors of the female reproductive system. Lymph nodes metastasis, the most common metastasis, which can be detected even in small-size tumor patients, results in worse prognosis. Therefore, it is of great significance to explore novel lymph nodes metastasis associated biomarkers, which can predict the prognosis and provide a good reference for clinical decision making in cervical carcinoma patients. However, systematic and comprehensive studies related to the key molecules in lymph node metastasis in cervical carcinoma patients are still absent.

**Methods:**

Transcriptome and clinical data of 307 cervical carcinoma patients were obtained from The Cancer Genome Atlas (TCGA). Then, survival of patients with and without lymph node metastasis was analyzed by Kaplan-Meier (K-M) curves. Differential expressed genes (DEGs) were detected between tumor and control samples using limma package and defined as lymph node metastasis related genes. Univariate and multivariate Cox regression analyses were carried out to screen robust prognostic gene signature. The risk score model and nomogram for predicting survival were constructed based on prognostic gene signature. The performance of the risk score model was evaluated by operating characteristic (ROC) curves. Based on risk score, patients were divided into low- and high- risk groups. DEGs, functional enrichment analysis and tumor microenvironment (immune infiltration and expressions of immune checkpoints) were detected in low- and high-risk groups.

**Results:**

A total of 103 lymph node metastasis-associated genes were identified. Univariate and multivariate Cox regression analyses identified TEKT2, LPIN2, FABP4 and CXCL2 as prognostic gene signature. The risk score model was constructed and validated in cervical carcinoma patients. 345 DEGs identified between high- and low-risk groups were significantly enriched into immune-related biological processes. Furthermore, we found that the immune infiltration and expressions of immune checkpoints were significantly different between low- and high-risk groups.

**Conclusion:**

Our study revealed that lymph node metastasis played an important role in the prognosis of cervical carcinoma patients. Furthermore, we established a risk score model based on lymph node metastasis related genes, which could accurately predict the survival of cervical carcinoma patients. Besides, our findings in tumor microenvironments of low- and high-risk groups improved our understanding of the relationship between lymph node metastasis related genes and cervical carcinoma.

**Supplementary Information:**

The online version contains supplementary material available at 10.1186/s12885-021-08945-8.

## Introduction

Uterine cervical cancer is a quite common female malignancy after thyroid cancer, with increasing trend of mortality in young females [1]. Importantly, a lot of cervical cancer patients may suffer from unpredicted risk of recurrence, metastasis and death. In clinical practice, surgery, chemotherapy and radiotherapy are applied for patients at different clinical stages. Unfortunately, the prognosis of some patients remain poor even after standard treatment [2], indicating the complicated etiology of cervical cancer. Therefore, prognosis associated factors and biomarkers are urgently needed for better clinical practice.

Lymph nodes metastasis is the most common phenomenon in cervical cancer metastasis and is an independent factor for poor prognosis of cervical cancer. Moreover, the staging system of cervical cancer have been revised based on lymph nodes metastasis in 2018 [3]. In our previous study, we confirmed that lymph nodes metastasis was significantly associated with the up-regulation of immune biomarkers. Mechanisms contributing to lymph nodes metastasis is very complex. Signal transduction by protein interaction and metabolism reprogramming may be involved in this process [4]. Therefore, comprehensive study on lymph nodes metastasis in cervical cancer using bioinformatic strategy will provide better understanding of this phenomenon and lead to the identification of promising biomarkers in predicting the prognosis of cervical cancer patients.

## Materials and methods

### Data collections based on TCGA

The RNA-seq profile and clinical information of 307 cervical carcinoma patients were downloaded from The Cancer Genome Atlas (TCGA) website (https://portal.gdc.cancer.gov/). The GTF annotation file was downloaded from the Ensembl Genome Browser (http://asia.ensembl.org/index.html) to convert the Ensembl gene ID into the gene symbol and extract the mRNA profile.

### Survival analysis of patients with and without lymph node metastases

Patients (*n* = 172) with cervical carcinoma were divided into non-lymph node metastasis (N0) and lymph node metastasis (N1) groups according to the presence or absence of lymph node metastasis after excluding patients with survival time less than 30 days and no information on lymph node metastases (Nx). Among these patients, 104 received radiotherapy, 84 received drug therapy and 107 received a combination of radiotherapy and drug therapy. Kaplan-Meier survival analysis was applied to compare the overall survival (OS) of cervical carcinoma patients in the N0 and N1 groups via R package ‘survival’.

### Differentially expressed genes

Differentially expressed genes (DEGs), which were defined as lymph node metastasis related genes, were identified between N0 and N1 groups with the threshold of absolute log2-fold-change (FC) more than 0.5 and *p* values less than 0.05 with R package ‘limma’. And the volcano plot and heatmap were used to demonstrate the gene expression patterns using R package ‘ggplot’ and ‘pheatmap’, respectively.

### Construction of the risk score model based on lymph node metastases associated prognostic signature

Cervical carcinoma patients were divided into a training set and a validation set in a ratio of 7:3, and the training set was used to construct a lymph node metastasis associated prognostic signature. The univariate Cox regression analysis was used to screen the genes significantly correlated with prognosis, and a forest plot was drawn via R package ‘survival’. Then, the genes with *p* value less than 0.05 were used to perform the multivariate Cox regression analysis to obtain robust gene signature (*p* < 0.05). We then calculated the risk score of each sample based on the expressions and regression coefficients of gene signature. The median risk score of all samples was used as the cut-off value to divide the patients into high- and low- risk groups.

### Assessment and validation of the risk score model based on lymph node metastases associated prognostic signature

Kaplan-Meier survival curves were used to compare the overall survival (OS) between high- and low- risk cervical carcinoma patients. Area under the curve (AUC) of receiver operating characteristic (ROC) curves were used to assess the sensitivity and accuracy of the risk score in predicting the OS of cervical carcinoma patients. At the same time, the same methods were carried out in the validation set to verify the replicability and stability of the lymph node metastases associated prognostic signature.

### Identification of independent prognostic factors in cervical carcinoma

In order to investigate the independent prognostic factor in cervical carcinoma, univariate and multivariate Cox regression analyses were utilized to evaluate the prognostic capability of risk score, age and N stage. And the factor with *p* value less than 0.05 was used as an independent prognostic factor for patients with cervical carcinoma.

### Construction and validation of nomogram for predicting survival

For the effective clinical application of the risk score, the nomogram for predicting 1-, 3- and 5-year survival rate of cervical carcinoma patients was constructed using R package ‘rms’.

### Functional enrichment analysis for high- and low- risk groups

Firstly, the DEGs were identified between the high- and low- risk groups using R package ‘limma’. The threshold value for DEGs screening was set to the absolute value of Log2FC more than 1 and *p* values less than 0.05. Next, the Gene Ontology (GO) analysis was performed to predict the the function of DEGs via R package ‘clusterProfiler’. The selection criterion for pathway enrichment results were *P* values less than 0.05.

### Immune cell infiltration

CIBERSORT deconvolution algorithm was used to accurately determine the infiltration of 22 immune cells in cervical carcinoma samples. The immune cell subtypes were characterized and quantified using gene expression signatures (LM22 files) which downloaded from the CIBERSORT website. And the samples with *p* values less than 0.05 were filtered.

### Statistical analysis

In our studies, all statistical analyses were performed in R (version 4.0.3), and p value less than 0.05 was considered statistically significant. The Wilcoxon test was used to compare differences between two groups. Kruskal-Wallis test was used to compare differences among multiple groups. Pearson’s correlation analysis was performed to calculate correlations among immune cells and correlations among gene signature and differentially distributed immune cells.

## Results

### Cervical carcinoma patients with non-lymph node metastases had a better prognosis

**Fig. 1:**
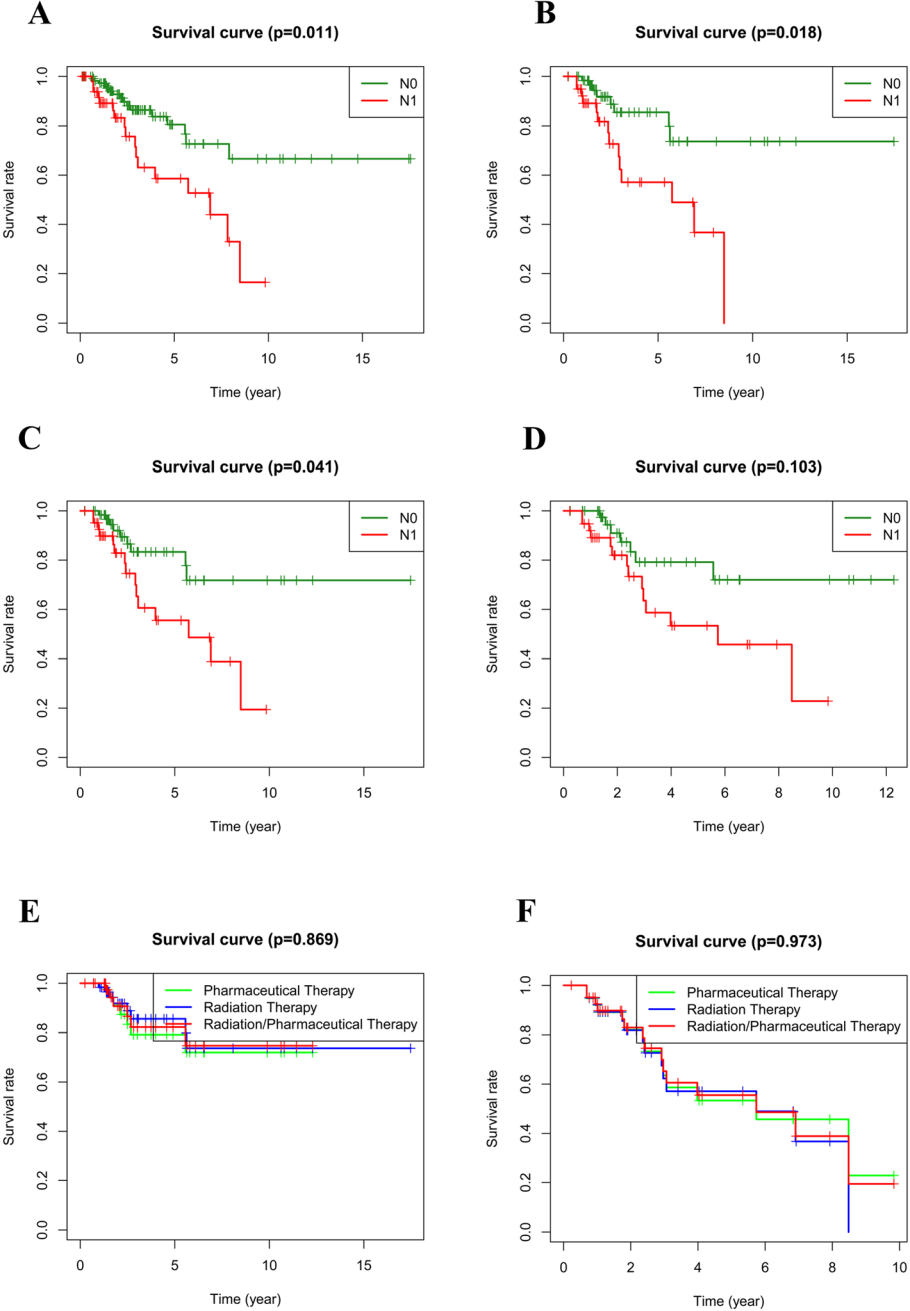
Survival analyses in selected cohorts. **A** K-M analysis of non-lymph node metastasis and lymph node metastasis groups; **B** K-M analysis of non-lymph node metastasis and lymph node metastasis patients treated by radiotherapy; **C** K-M analysis of non-lymph node metastasis and lymph node metastasis patients treated by radiotherapy and drug therapy; **D** K-M analysis of radiotherapy patients treated by drug therapy; **E** K-M analysis of patients with non-lymph nodes metastasis treated by different therapies; **F** K-M analysis of patients with lymph nodes metastasis treated by different therapies

The corresponding clinicopathological characteristics of patients included in this analysis were summarized in Table 1. Kaplan-Meier survival analyses results showed that patients with non-lymph node metastases (N0) enjoy significantly better prognosis than patients with lymph node metastases (N1) (*P* = 0.011, Fig. 1A). In addition, patients with N0 who received radiotherapy or a combination of radiotherapy and drug therapy had a significantly better prognosis than those with N1 (*P* < 0.05, Fig. 1B and C), while there was no significant difference between patients in N1 and N0 patients who received drug therapy only (*P* = 0.103, Fig. 1D) regarding to prognosis. We found that no significant difference among patients treated by different therapies in either N0 or N1 groups (*P* > 0.05, Fig. 1E and F) regarding to prognosis. These results indicated that lymph node metastasis was significantly correlated with patients’ survival. Thus, lymph node metastasis-related genes might serve as prognostic biomarkers for patients with cervical carcinoma.

**Table 1:** Clinicopathological characteristics of patients included in this analysis

		N		
	Total (***N***=95)	N0 (***N***=73)	N1 ***N***=22)	***P*** value
**Age**(**years**)				
≤**50**	63 (66.3%)	47 (64.4%)	16 (72.7%)	0.639
>**50**	32 (33.7%)	26 (35.6%)	6 (27.3%)	
**Vital status**				
**Alive**	78 (82.1%)	62(84.9%)	16 (72.7%)	0.321
**Dead**	17 (17.9%)	11(15.1%)	6 (27.3%)	
**M**				
**M0**	92 (96.8%)	72 (98.6%)	20 (90.9%)	0.263
**M1**	3 (3.2%)	1 (1.4%)	2 (9.1%)	
**T**				
**T1**	70 (73.7%)	55 (75.3%)	15 (68.2%)	0.0512
**T2**	21 (22.1%)	17 (23.3%)	4 (18.2%)	
**T3**	2 (2.1%)	0 (0%)	2 (9.1%)	
**T4**	2 (2.1%)	1 (1.4%)	1 (4.5%)	
**Treatment type**				
**no,no**	32 (33.7%)	31 (42.5%)	1 (4.5%)	<0.001
**no,yes**	12 (12.6%)	12 (16.4%)	0 (0%)	
**yes,no**	3 (3.2%)	2 (2.7%)	1 (4.5%)	
**yes,yes**	48 (50.5%)	28 (38.4%)	20 (90.9%)	
**Stage**				
**I**	66 (69.5%)	54 (74.0%)	12 (54.5%)	<0.001
**II**	16 (16.8%)	15 (20.5%)	1 (4.5%)	
**III**	9 (9.5%)	2 (2.7%)	7 (31.8%)	
**IV**	4 (4.2%)	2 (2.7%)	2 (9.1%)	

*Treatment type: no,no: no chemotherapy nor radiotherapy

no, yes: no chemotherapy, but treated by radiotherapy

yes, no: treated by chemotherapy, but no radiotherapy

yes, yes: treated by both chemotherapy and radiotherapy

### Identification of 103 lymph node metastasis-related genes

**Fig. 2:**
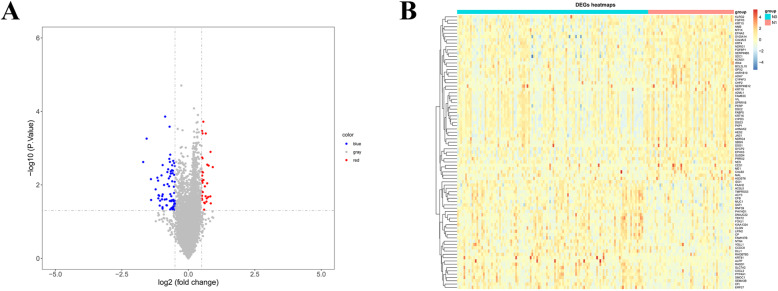
Identification of Lymph node metastasis-related genes. **A** DEGs between non-lymph node metastasis (N0) and lymph node metastasis (N1) groups were shown in the volcano plot. **B** 33 up-regulated and top 50 down-regulated genes were shown in the heatmap

Thereafter, we identified a total of 103 differentially expressed genes (DEGs), including 33 up-regulated DEGs and 70 down-regulated DEGs (Supplementary Table S1). The DEGs were visualized by volcano plot, with red solid dots representing up-regulation genes, blue solid dots representing down-regulation genes and grey solid dots representing genes that are not significantly different (Fig. 2A). Furthermore, 33 up-regulated genes and top 50 down-regulated genes in each sample were shown in the heatmap (Fig. 2B).

### Construction of the risk score model based on four lymph node metastasis-associated genes

**Fig. 3:**
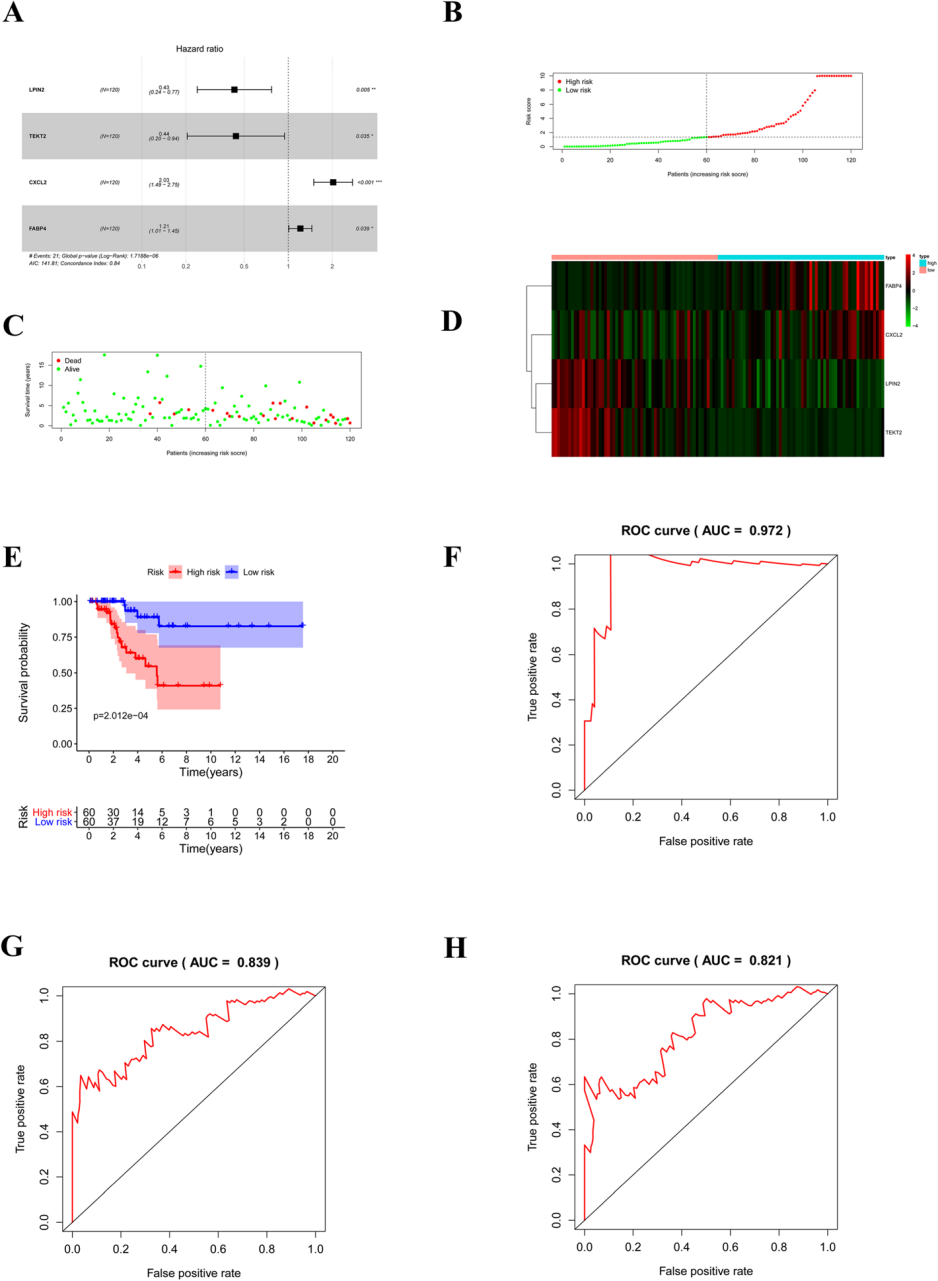
Construction of the risk score model in the training set. **A** Four lymph node metastasis-associated genes were identified in multivariate Cox regression analysis; **B** Patients were divided into high- and low-risk groups according to the median of the risk score; **C** More dead patients were observed with higher risk score; D Heatmap showed the expressions of prognostic gene signature in high- and low-risk groups. **E** K-M analysis of high- and low-risk groups **F-H** ROC curves of 1-, 3- and 5-year survival prediction of cervical carcinoma patients

A total of 120 cervical carcinoma patients in TCGA were used to construct the prognostic signature. We found that 11 genes (CXCL2, SERPINB5, THBD, LPIN2, PTP4A1, FABP4, TEKT2, BCL2L10, ME1, IRX4, and SDC1) were significantly associated with survival in patients with cervical carcinoma according to the results of univariate Cox regression analysis of 103 DEGs (Supplementary Table S2, P < 0.05). Then, further multivariate Cox regression analysis identified LPIN2, TEKT2, CXCL2, and FABP4 as gene signature to construct the risk score model (Supplementary Table S3, Fig. 3A). Subsequently, the risk score of each sample was calculated based on the expression and regression coefficient of genes acquired from the multivariate Cox regression analysis as following: Risk score = (− 0.8468 × expression level of LPIN2) + (− 0.8232 × expression level of TEKT2) + (0.7068 × expression level of CXCL2) + (0.1906 × expression level of FABP4). Thereafter, we divided the patients into high- and low- risk groups according to the median of risk score and plotted the risk curve **(**Fig. 3B). Corresponding to the risk curve, more dead patients were observed while risk score increasing (Fig. 3C). And the gene expression heatmap showed that FABP4 and CXCL2 were up-regulated in high-risk group while LPIN2 and TEKT2 were down-regulated (Fig. 3D). Furthermore, the Kaplan-Meier survival curves indicated that patients in the high-risk group have worse prognosis (*P* = 2.012e-04, Fig. 3E), and the ROC revealed a strong confidence of the risk score model in predicting survival of cervical carcinoma patients and the AUCs for predicting 1, 3, and 5-year survival were 0.972, 0.839, and 0.821, respectively **(**Fig. 3F). The result of Harrels c-index measure showed that the index values before and after correction were 0.520 and 0.509 respectively (Table 2).

**Table 2:** The c-index value in the training set and testing set

C-index	Training set	Testing set
Before correction	0.520	0.543
After correction	0.509	0.522

### Validation of the risk score model

**Fig. 4:**
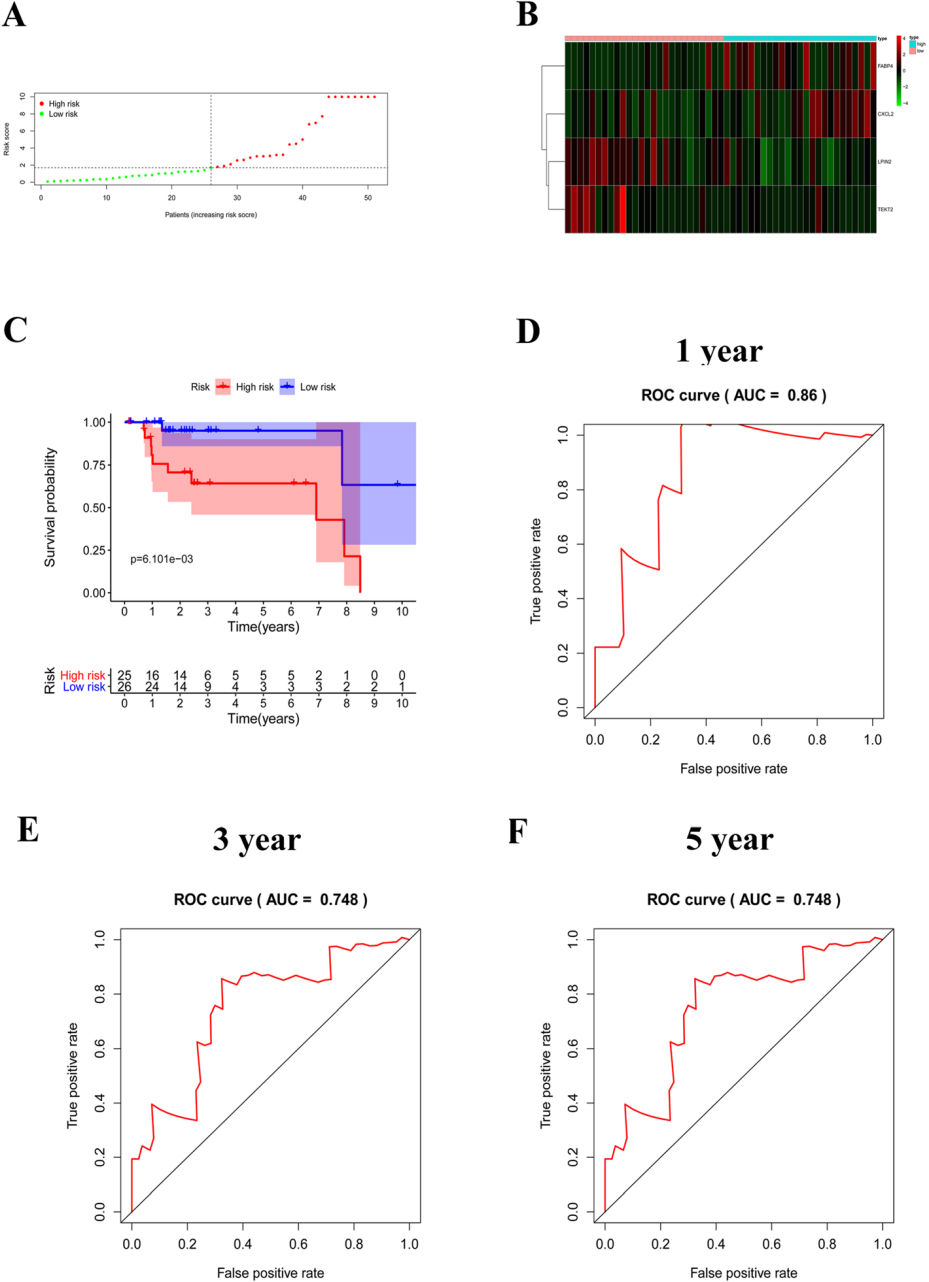
Validation of the risk score model in the validation set. **A**. Patients were divided into high- and low-risk groups according to the median of the risk score; **B**. Heatmap showed the expressions of prognostic gene signature in high- and low-risk groups. **C** K-M analysis of high- and low-risk groups; **D-F** ROC curves of 1-, 3- and 5-year survival prediction of cervical carcinoma patients

We further validated the risk model in 51 patients from internal validation set. Similarly, patients were divided into low- and high-risk groups according to the median of risk score **(**Fig. 4A). At the same time, the expression trends of FABP4, CXCL2, LPIN2 and TEKT2 were consistent with results observed in the training set (Fig. 4B). In addition, Kaplan-Meier survival curves showed that patients in high-risk group also had worse prognosis than those in low-risk group (*P* = 6.101e-03, Fig. 4C). The AUCs of ROC curves for predicting 1-, 3- and 5-year survival of cervical carcinoma patients in the validation set were 0.86, 0.748, and 0.748 (Fig. 4D-F). These results indicated that the risk score model had high accuracy in predicting survival of cervical carcinoma patients.

### The relationship among the risk score and clinical characteristics

**Fig. 5:**
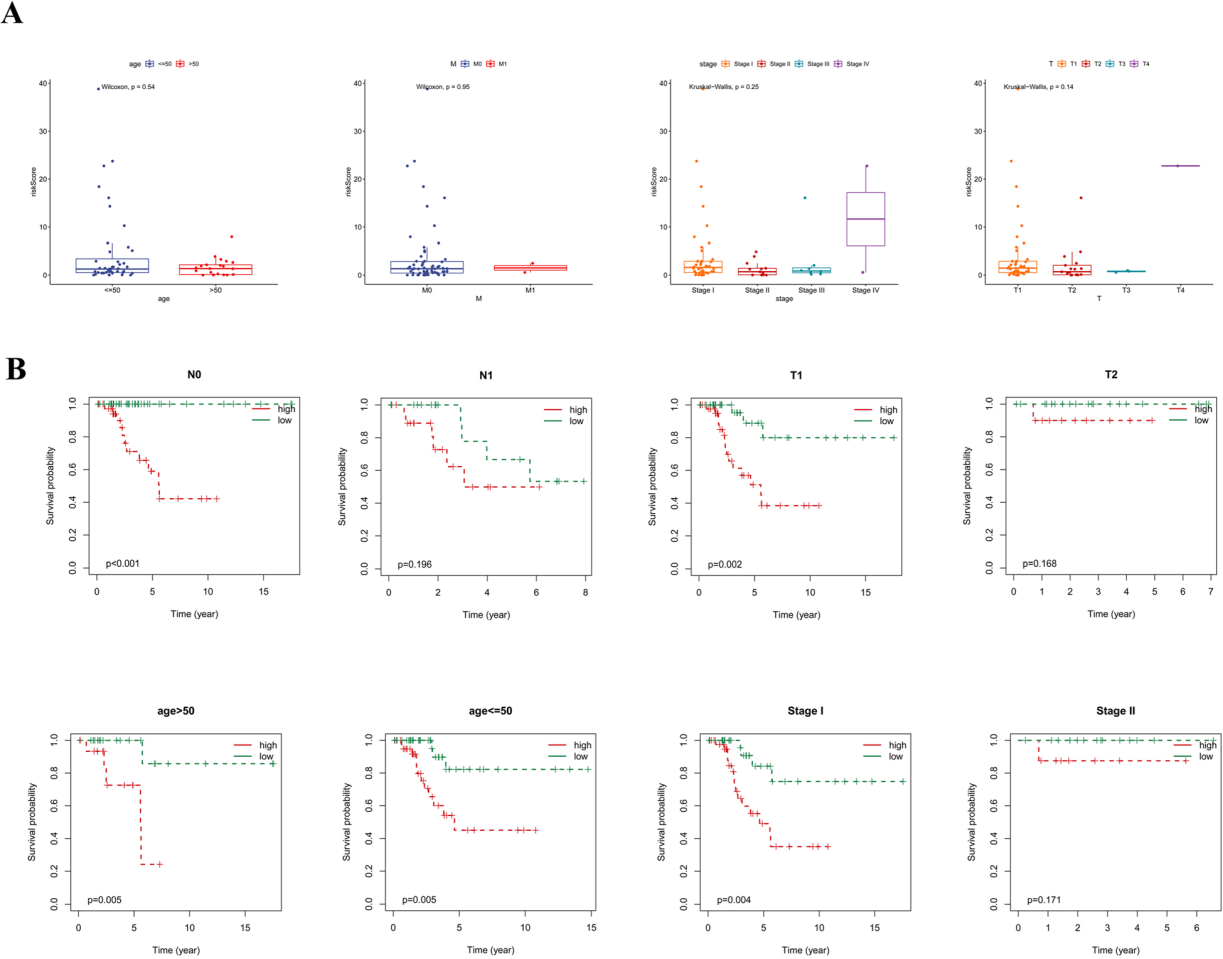
Association between the risk signature and clinical characteristics. **A**. Comparison of risk scores among subgroups divided by age, M stage, T stage and FIGO stage. **B**. K**-**M analysis of high- and low-risk groups under different clinical features

Furthermore, we investigated the relationship among the risk score and clinical characteristics. We found that there was no significant difference of risk score among groups divided by age, M stage, T stage and FIGO stage (*P* > 0.05, Fig. 5A). K-M curves showed that patients in high risk group with clinical features including age > 50, age ≤ 50, N0, Stage I, and T1 had a worse prognosis than those in low-risk group (*P* < 0.05, Fig. 5B), while there was no significant difference in prognosis between patients in low- and high-risk groups characterized by N1, Stage II, and T2 clinical features (*P* > 0.05, Fig. 5B).

### Risk score is an independent prognostic predictor of survival in patients with cervical carcinoma

**Fig. 6:**
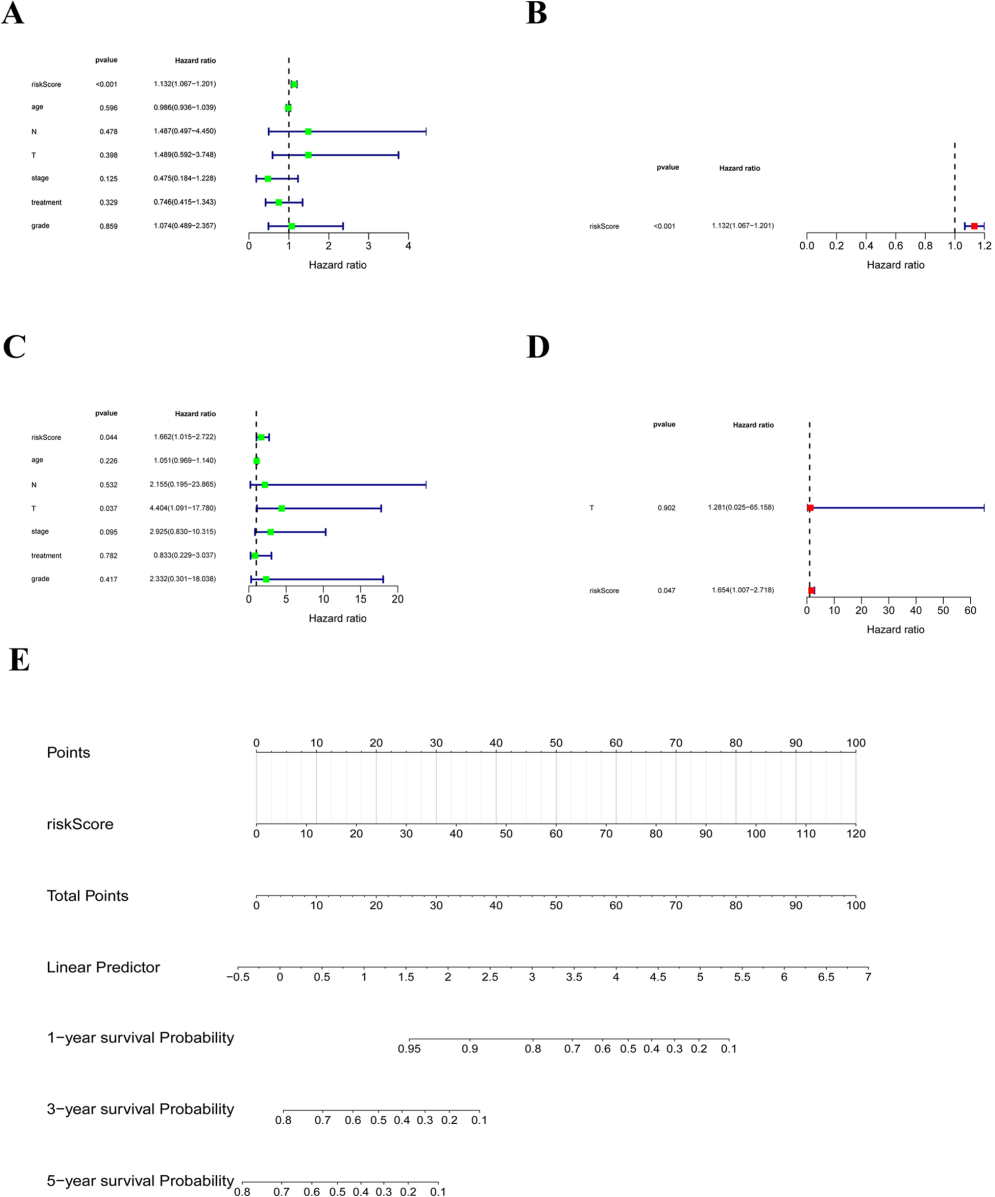
Construction of the nomogram. **A-B** Risk score is a independent prognosis factor in both univariate and multivariate Cox regression analyses in training set. **C-D** Risk score is a independent prognosis factor in both univariate and multivariate Cox regression analyses in validation set. **E** Nomogram for survival prediction in cervical carcinoma

Then, we investigated whether the risk score was an independent prognostic factor in cervical carcinoma. Firstly, we included risk score, age, T stage, N stage, FIGO stage grade and treatment to conduct univariate Cox regression analysis in the training set. We found that the risk score was significantly associated with prognosis (*P < 0.001*, Fig. 6A). Further multivariate Cox regression analysis showed that the risk score remained strongly associated with prognosis (*P* < 0.001, Fig. 6B), revealing that the risk score was an independent prognostic factor in cervical carcinoma. Similar results were obtained in the validation set **(**Fig. 6C and D). Next, we constructed a nomogram based on the risk score to predicting 1-, 3- and 5-year survival of cervical carcinoma patients (Fig. 6E).

### Lymph node metastasis-related genes may regulate cervical carcinoma via immune-related biological processes

**Fig. 7:**
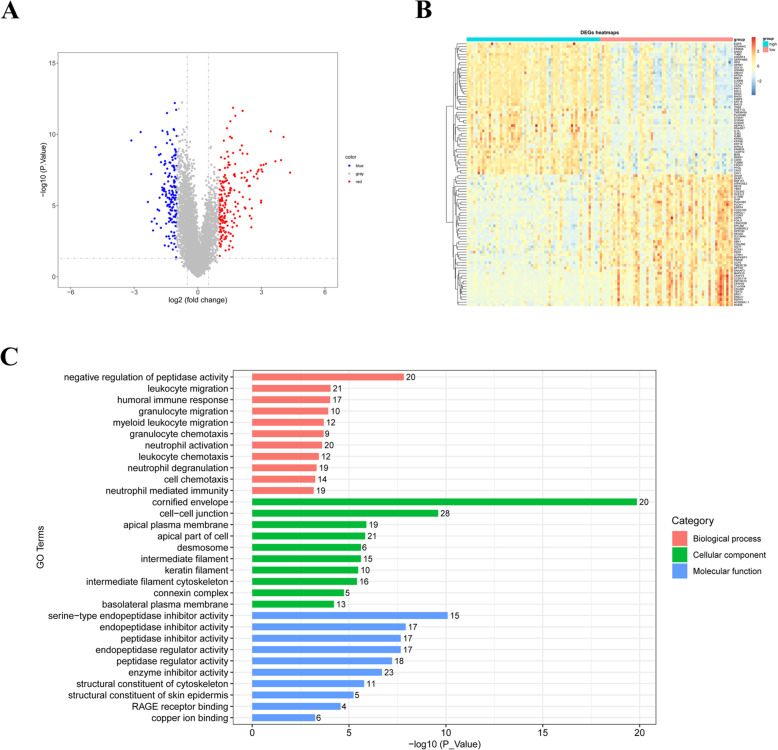
Functional enrichment analysis of DEGs between high- and low-risk groups. **A** DEGs between high- and low-risk groups were shown in the volcano plot. **B** Top 50 up-regulated and top 50 down-regulated genes were shown in the heatmap. **C** GO enrichment analysis of DEGs

We further explored the mechanisms underlying the differences in survival between high- and low- risk groups. A total of 345 DEGs were identified with |log2(fold change)| > 1 and *P* value < 0.05 (Supplementary Table S4, Fig. 7A, B). Then, GO enrichment analysis including biological processes, cellular components and molecular functions, were performed to analyze the biological functions of these DEGs. The results showed that DEGs were enriched in biological processes and cellular components closely related to the immune system, such as ‘leukocyte migration’(*P* = 8.97E-05), ‘humoral immune response’(*P* = 9.45E-05), ‘myeloid leukocyte migration’(*P* = 0.0002), ‘granulocyte chemotaxis’(*P* = 0.0002), ‘neutrophil activation’(*P* = 0.0002), and ‘neutrophil mediated immunity’(*P* = 0.0007), etc. (Supplementary Table S5, Fig. 7C).

### The immune landscape of the high- and low-risk groups

**Fig. 8:**
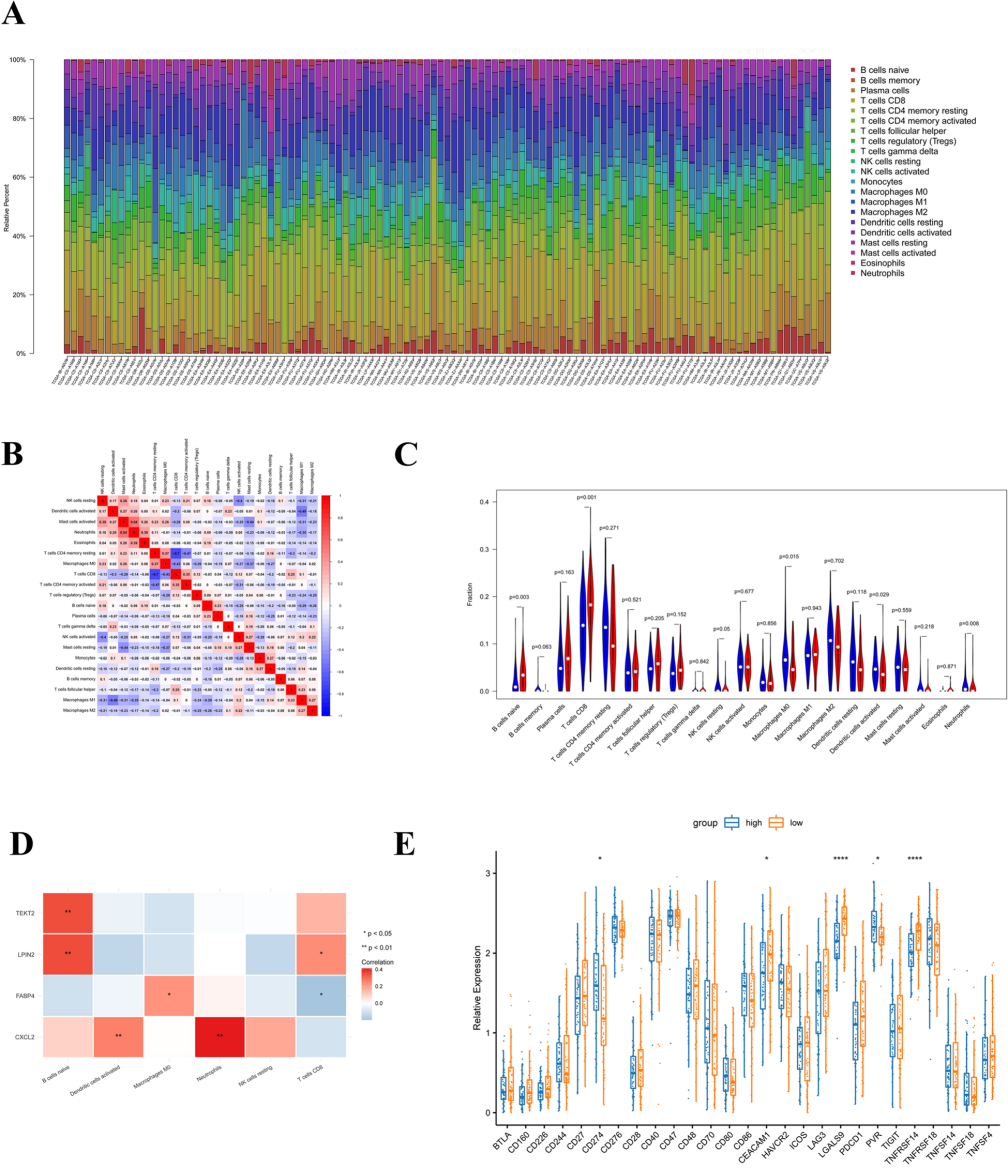
Tumor microenvironment characteristics in low- and high-risk groups. **A** Proportions of different immune cell types in selected samples. **B** Correlations between different immune cell types. **C** Comparison of immune infiltration between high- and low -risk group (Blue: high risk group; Red: low risk group). **D** Correlations among prognostic gene signature and differentially distributed immune cells. **E** The expressions of immune checkpoint molecules in high- and low-risk groups

The above findings led us to hypothesize that the prognostic signature might be related to the tumor microenvironment of cervical carcinoma. Firstly, the proportions of 22 immune cell types in 171 samples were estimated based on the LM22 signature files with CIBERSORT [5]. The abundance of 22 immune cells in 113 significant samples (*P* < 0.05) were shown in Fig. 8A. We found that there were no naive CD4 T cells distributed in either low- or high-risk group. Meanwhile, the correlations between 21 immune cell types were analyzed by Pearson correlation. And we found that resting memory CD4 T cells showed the highest negative correlation with CD8 T cells (cor = − 0.7), followed by activated memory CD4 T cells (cor = − 0.47). Besides, M1Macrophages also showed a high negative correlation with activated dendritic cells (cor = − 0.49). And activated mast cells showed a strong positive correlation with neutrophils (cor = 0.54) **(**Fig. 8B). Moreover, R package ‘vioplot’ was used to calculate the differences in immune cell types between high- and low-risk groups. We observed that the distributions of naive B cells, CD8 T cells, resting NK cells, M0 Macrophages, activated dendritic cells, and neutrophils were significantly different between two groups (*P* < 0.05, Fig. 8C).

Next, we analyzed the relationship among four prognostic gene and differentially distributed immune cells (Table S6 and Fig. 8D). The results showed that TEKT2 was positively correlated with naive B cells (*p* < 0.01). LPIN2 had strong positive correlations with naive B cells (p < 0.01) and CD8 T cells (*p* < 0.05). FABP4 was positively correlated with M0 macrophages (p < 0.05), but negatively correlated with CD8 T cells (p < 0.05). As for CXCL2, it was found to be positively correlated with activated dendritic cells (p < 0.01) and neutrophils (p < 0.01).

Furthermore, we detected the expression of immune checkpoint molecules correlated with immune response in low- and high-risk groups. We observed that CD274, CEACAM1, LGALS9, PVR and TNFRSF14 were significantly different **(*P*** **< 0.05,** Fig. 8E) between two groups, providing a novel insight into immunotherapy for cervical carcinoma patients.

## Discussion

In recent years, NGS enable us to have a broader and deeper view in tumor. Numerous researches have validated the relationship between lymph nodes metastasis and poor prognosis [6–8]. Lymph nodes metastasis is quite common in cervical cancer. Recent years, researchers have revealed some possible mechanisms involved in lymph nodes metastasis in cervical cancer [9–11],indicating that lymph nodes metastasis is a complicated progress deserving more attention. However, comprehensive analysis about the genes involved in this process is still absent. Therefore, we integrated RNA-Seq data and clinical information of cervical cancer patients in TCGA database. 103 genes associated with lymph nodes metastasis were identified. Prognostic gene signature LPIN2, TEKT2, CXCL2, and FABP4 was obtained from these 103 genes by univariate and multivariate Cox analyses. Then, we constructed a risk score model based on LPIN2, TEKT2, CXCL2, and FABP4, which had high accuracy in predicting the prognosis of cervical cancer patients. Moreover, we found that Lymph nodes metastasis may influence the prognosis of cervical cancer patients via tumor microenvironment, as evidenced by different immune cells infiltration and expressions of immune checkpoints between low- and high-risk groups.

TEKT2, LPIN2, CXCL2, and FABP4 were found to be associated with lymph nodes metastasis and identified as prognostic gene signature in our research. TEKT2 belongs to Tektin family, which is important in cytoskeleton formation [12]. Several studies have reported its role in spermatogenesis [13, 14]. However, its role in cervical cancer remains unclear. Destroyed cytoskeleton may inhibit cell migration and invasion in cervical cancer [15]. Proteomic analysis in cervical cancer cell lines revealed aberrant expression of cytoskeleton associated protein [16]. Therefore, we speculated that TEKT2 may regulate cervical cancer by affecting cytoskeleton in cervical cancer cells, which needs further study in the future. Moreover, we found TEKT2 is a protective factor, which is supported by its higher expression in low-risk group. Another protective factor in cervical cancer found in the current study was LPIN2. LPIN2 can regulate MAPK activation, which mediates synthesis of pro-IL-1β during inflammasome priming [17]. It has reported that IL-1β inflammatory response could inhibit breast cancer cell metastasis [18]. Thus, LPIN2 may affect the prognosis of patients with cervical cancer via IL-1β. CXCL2 and FABP4 have been intensively studied in malignancies. These two risky factors regulate the tumorigenesis via complicated mechanisms such as chemokine releasing and lipid metabolism [19–23]. In cervical cancer, CXCL2 may promote tumor growth and angiogenesis and NF-κB pathway is involved [24]. FABP4 may promote epithelia epithelial-mesenchymal transition (EMT) by activating AKT pathway [25], which supports its upregulation in high-risk group in our research. More solid experiments are needed to clarify the detailed functions and associated molecular mechanisms of these genes in cervical cancer. We constructed a risk score model based on the expressions and coefficients of these 4 genes. Then we divided these patients into high-risk and low-risk groups. To better understand the roles of these 4 lymph nodes metastasis associated genes in prognosis, we performed GO enrichment analysis of 345 DEGs between low- and high-risk group. We found that the DEGs were mainly enriched into immune related biological processes, one of which was humoral immune response (Fig. 7C). Humoral immune response is often observed in B cell associated immunity and plays an important role in virus or bacteria defection. Cervical cancer is significantly associated with HPV infection. Humoral immune response against HPV is an important process and it could allow the determination of the infection stage (transient, latent, persistent) and the stage of the disease [26]. Humoral immune response will facilitate antigen cross-presentation and activation of T cells, which have led to better application of HPV vaccine [27, 28].

All results above indicated that prognostic signature had a strong relationship with immunity. Immune cells are important parts for immune system and tumor infiltrating lymphocytes (TILs) could be a marker for prognosis prediction [29]. Therefore, we compared the distribution of 22 types immune cells between high and low-risk groups, and analyzed the relationship between differentially distributed immune cells and prognostic signature. Interestingly, we found that the relationship between the abundance of neutrophils and the expression of CXCL2 was the most significant. In a previous study, CXCL2 signals can induce neutrophils aging [30], which supports significant relationship between CXCL2 and neutrophils found in our study (Fig. 8D). Neutrophils can infiltrate solid tumors at different extent. In some types of human tumors, high infiltration of tumor-associated neutrophils (TANs) is associated with poor prognosis [31, 32]. In cervical cancer, TANs density is an independent prognostic factor for shorter survival in those who were treated by definitive radiotherapy [33]. In following research, TANs even promotes radio- resistance in cervical cancer and MAPK pathway maybe involved [34]. Therefore, we supposed that CXCL2 may lead to activation of neutrophils signaling and result in poor prognosis in cervical cancer, which deserves further research.

Finally, we compared the expressions of checkpoints in high- and low-risk groups. The expressions of five checkpoint molecules were significantly different. Among them, CD274 (also named PD-L1),which was up-regulated in high-risk group, attracted our attention. PD-L1 antibody is quite promising in cervical cancer treatment [35]. In our previous study, PD-L1expression promotes lymph nodes metastasis in cervical cancer [4]. Herein, we supposed that prognostic signature may regulate lymph nodes metastasis via affecting the expression of PD-L1.

In summary, for the first time, our research revealed 4 genes associated with lymph nodes metastasis in cervical cancer and provided a prognostic prediction model based on these 4 genes. Besides, we explored the potential immune related mechanisms of these 4 genes in regulating cervical cancer. Our study has some limitations due to the absence of clinical validation and limited populations of cervical cancer patients in TCGA database. Our findings enrich our understanding of cervical cancer etiology, which will contribute to good clinical practice in immunotherapy and future target therapy research.

## Supplementary Information

**Additional file 1.**

**Additional file 2.**

**Additional file 3.**

**Additional file 4.**

**Additional file 5.**

## Data Availability

This study obtained open data from the TCGA database. (https://cancergenome.nih.gov/).
